# Associations of smoking behavior with lifestyle and mental health among Japanese dental students

**DOI:** 10.1186/s12909-018-1365-1

**Published:** 2018-11-16

**Authors:** Yuko Fujita, Kenshi Maki

**Affiliations:** All in the Division of Developmental Stomatognathic Function Science, Department of Health Promotion, Kyushu Dental University, 2-6-1 Manazuru, Kokurakita-ku, Kitakyushu, 803-8580 Japan

**Keywords:** Dental students, Smoking behavior, Life style, Mental health

## Abstract

**Background:**

Smoking is a major risk factor for cancer and cardiovascular disease. However, mental stress leads to smoking in dental students. We believe that dentists, as health professionals, should set an example for the public. Therefore, this study determined the prevalence of and factors associated with regular smoking among Japanese dental students. We also surveyed their attitudes regarding smoking cessation and how to counsel smokers about quitting.

**Methods:**

We collected demographic and behavioral data from 453 students at Kyushu Dental University, and evaluated their mental health with the 12-item General Health Questionnaire (GHQ 12). We also asked them nine questions related to their intentions to counsel smokers about quitting. A multivariate binary logistic regression analysis was used to identify factors associated with smoking.

**Results:**

Fifty-two (11.5%) of the dental students smoked. Univariate analyses indicated that male gender, higher academic year, greater number of times eating out per day, alcohol consumption, prevalence of skipping breakfast, poor health, and poor sleep habits were significantly associated with regular smoking. Regular smokers were less likely to have GHQ 12 scores ≥4. On multivariate analysis, male gender (OR = 5.449, 95% CI = 1.851–16.040), sixth year students (OR = 21.971, 95% CI = 1.686–286.290), eating out two or more times a day (OR = 2.492, 95% CI = 1.165–5.331), drinking alcohol three or more times per week (OR = 9.484, 95% CI = 3.335–26.970), and GHQ 12 score ≥ 4 (OR = 0.339, 95% CI = 0.136–0.845) were significantly associated with regular smoking. Overall, 50.1% of the non-smokers and 71.2% of the regular smokers responded that patients’ chances of quitting smoking are not increased when a dentist advises them to quit.

**Conclusions:**

Regular smoking was strongly associated with male gender, higher academic year, alcohol consumption, and higher frequency of eating out per day. Mental health status among regular smokers was better than that among non-current smokers. Furthermore, we found that more than half of dental students have inadequate attitudes to advise their patients to quit smoking. It is necessary to develop educational programs regarding smoking for dental students.

## Background

Smoking is a major risk factor for cancer [1–3] and cardiovascular disease [4], and both smoking and drinking alcohol are health hazards. A recent World Health Organization study of the global tobacco epidemic showed that 21% of adults globally, or 950 million men and 177 million women, were current smokers in 2013. Smoking prevalence is highest in high-income countries, with 25% of adults being current smokers in 2013 [5]. In Japan, the National Health and Nutrition Survey showed that the prevalence of current cigarette smoking among Japanese adult males decreased gradually from 39.9% in 2006 to 30.2% in 2016 [6]. In the USA, cigarette smoking among adult males declined from 23.9% in 2005 to 18.8% in 2014 [7]. These data suggest that the prevalence of smoking in Japanese men is high among developed nations.

The reported prevalence of current smoking among Japanese male dentists was 27.1% in 2008 [8], which is significantly higher than the rate among Japanese male physicians (15.0%) [9]. It has been reported that the smoking rate among physicians is declining in Japan [10], while changes in prevalence of smoking among dentists are still unclear. In addition, no information is available regarding the attitudes of Japanese dentists toward smoking cessation in recent years. Several studies have shown that many dental students suffer from psychological stress due to their many examinations and personal problems [11, 12]. A recent study showed that stress from the heavy course load was the main reason that dental students initiated smoking [13]. A few studies have suggested that the nicotine contained in tobacco has antidepressant-like effects in depressed patients [14] and small animal models [15]. Therefore, we hypothesized that students with mental health disturbances depend on the use of tobacco to improve their condition. However, dentists have a responsibility to provide public awareness of smoking prevention and cessation, and to facilitate improvements in public health. We also believe that dentists as health professionals should serve as examples to the public. However, there is no information regarding the associations between smoking behavior and lifestyle and mental health or attitudes regarding smoking intervention among Japanese dental students.

This study determined the prevalence of and factors associated with regular smoking among Japanese dental students and surveyed their attitudes toward smoking cessation and counseling smokers about quitting.

## Methods

This study was approved by the Human Investigations Committee of Kyushu Dental University (14–35) and all subjects provided written informed consent before participating.

A survey was completed by 453 dental students (200 women, 253 men) in six dental classes at Kyushu Dental University in April 2015. These students entered dental university after graduating from high school in April 2015. The first and second years of this university program provide general and preclinical education. The third and fourth years are also preclinical, but at the end of fourth year, students can take common achievement tests, including computer-based testing and the Objective Structured Clinical Examination. In the fifth and sixth years, students undertake clinical clerkship at the university hospital.

We planned a study with 40 regular smokers and 413 non-current smokers. Prior data indicated that 63% of non-current smokers were male [16]. With a true within-stratum odds ratio for regular smoking in males relative to females of 3.5, we would be able to reject the null hypothesis that this odds ratio equals 1 with probability (power) 0.882. The type I error probability associated with this test of the null hypothesis was 0.05. We used an uncorrected chi-squared statistic to evaluate this null hypothesis.

The survey solicited the following information: demographic characteristics (gender, age, and academic year), body mass index, current and past smoking habits, residence, eating habits, health status, physical activity, participation in club activities, sleep, alcohol use, participation in smoking cessation education, and smokers in respondents’ families. Mental health was assessed using the 12-item General Health Questionnaire (GHQ 12) Japanese version. The validity and reliability of GHQ12 have been previously confirmed [17, 18]. GHQ 12 responses were scored as 0 or 1, and the total score ranged from 0 to 12 points [19]. We used the cutoff of 4 points arrived at by Guthrie et al. [20] in their investigation of stress and burnout in medical students. High scores indicate low mental health status.

The participants were classified as non-current smokers and regular smokers. Regular smokers were those who answered “daily” or “sometimes” to the question on current smoking status in the last month; all others were defined as non-current smokers.

We evaluated whether the participants had professional responsibilities associated with smoking intervention using nine questions on professional responsibility adapted from a study of Japanese medical students [16]. One point was added when a suitable response was given to each question, and the sum of suitable responses was the individual professionalism score. All questionnaires were applied in Japanese. Factor analyses were applied to assess the validity of the questionnaire on professional responsibility [21]. For exploratory factor analysis, principal factor analysis with Varimax rotation was performed. Kaiser’s criterion (i.e., eigenvalues > 1.0) and a visual examination of scree plots were used to determine the number of components to retain [22–24]. Following principal factor analysis, confirmatory factor analysis was performed to explore the valid factor structure. The goodness-of-fit index (GFI), adjusted GFI (AGFI), comparative fit index (CFI), and root-mean-square error of approximation (RMSEA) were used as indices of conformity. In general, GFI, AGFI, and CFI values of ≥0.90 indicate good fits [25, 26]. In addition, the RMSEA was interpreted as follows: good, ≤ 0.05; and acceptable, ≤ 0.08 [27]. Furthermore, Cronbach’s alpha coefficient was used as an index of internal consistency of the questionnaire, with α ≥ 0.7 accepted as evidence of good internal consistency [28].

### Statistical methods

When appropriate, the chi-squared test or Fisher’s exact test was used to compare categorical variables between non-smokers and regular smokers. The independent *t*-test was used to compare the means of continuous variables. A multivariate binary logistic regression analysis was used to identify factors associated with smoking. *P*-values < 0.05 indicated statistical significance. In the binary logistic regression analysis, current smoking status was considered to be the dependent variable, and independent variables that were significant in the univariate analyses were included. Categorical variables were coded appropriately before they were entered into the model. The adjusted odds ratios (ORs) for smoking and their 95% confidence intervals (CIs) were calculated. All statistical analyses were performed using SPSS for Windows ver. 23.0 (IBM Japan, Tokyo, Japan).

## Results

As the results for validity and reliability of the questionnaire on professional responsibility, from Kaiser’s criterion, three factors had eigenvalues > 1, which together accounted for 55.2% of the variance in the total scores. The results of confirmatory factor analysis showed goodness-of-fit of the three-factor structure models in the questionnaires as follows: GFI = 0.947, AGFI = 0.910, CFI = 0.954, and RMSEA = 0.062. With regard to internal consistency of the questionnaire, Cronbach’s alpha coefficients of the scale and its three subscales were 0.701–0.853.

Table 1 summarizes the demographic characteristics of the 453 participants (200 women, 253 men) who completed the survey. The mean response rate of all students was 78.6%.

**Table 1:** Participant characteristics

Variable	Female (%)	Male (%)	Total (%)
Participants	200 (44.2)	253 (55.8)	453 (100)
Age (years)			
mean ± SD	21.69 ± 2.32	22.38 ± 2.58	22.07 ± 2.49
18–20	68 (34.0)	67 (26.5)	135 (29.8)
20–22	84 (42.0)	110 (43.5)	194 (42.8)
23–26	39 (19.5)	54 (21.3)	93 (20.5)
≥ 27	9 (4.5)	22 (8.7)	31 (6.8)
Academic year			Responses (%)
1st	34 (17.0)	42 (16.6)	76/97 (78.4)
2nd	40 (20.0)	46 (18.2)	86/103 (83.5)
3rd	35 (17.5)	34 (13.4)	69/97 (71.1)
4th	39 (20.5)	37 (15.0)	76/96 (79.2)
5th	22 (11.0)	46 (18.2)	68/87 (78.2)
6th	30 (15.0)	48 (19.0)	78/96 (81.3)
Ever smoked			
Never	183 (91.5)	163 (64.4)	346 (76.4)
Yes	17 (8.5)	90 (35.6)	107 (23.6)

*SD* standard deviation

Table 2 shows the results of the assessment of smoking habits according to demographics. Overall, 11.5% (*n* = 52) of respondents were regular smokers. The prevalence of regular smokers was significantly higher among men than among women (*p* = 0.000). Age and academic year were significantly higher among regular smokers than among non-current smokers (both *p* = 0.000).

**Table 2:** Assessment of smoking habits according to demographic characteristics

	Non-current smokers (%)	Regular smokers (%)	*χ* ^2^	*p*-value
Participants (*n* = 453)	401 (88.5)	52 (11.5)		
Gender				
Female	195 (48.6)	5 (9.6)		
Male	206 (51.4)	47 (90.4)		
			28.413	0.000^a^
Age (years)				
mean ± SD	21.88 ± 2.44	23.54 ± 2.39	–	0.000^b^
18–20	130 (32.4)	5 (9.6)		
21–23	168 (41.9)	26 (50.0)		
24–26	81 (20.2)	12 (23.1)		
≥ 27	22 (5.5)	9 (17.3)		
			18.324	0.000^a^
Academic year				
1st	75 (18.7)	1 (1.9)		
2nd	80 (20.0)	6 (11.5)		
3rd	62 (15.5)	7 (13.5)		
4th	69 (17.2)	7 (13.5)		
5th	53 (13.2)	15 (28.8)		
6th	62 (15.5)	16 (30.8)		
			23.702	0.000^a^
Body Mass Index (kg/m^2^)				
mean ± SD	20.85 ± 3.92	21.91 ± 2.55	–	0.070^b^
< 18.5	43 (14.7)	3 (6.1)		
18.5–24.9	216 (73.7)	41 (83.7)		
≥ 25.0	34 (11.6)	5 (10.2)		
			2.909	0.234^a^

*SD* standard deviation. ^a^ Chi-square test, ^b^*t* test

Table 3 summarizes the assessment of smoking habits according to behavioral variables and mental health. Smokers were more likely to skip breakfast twice or more a week, eat out once or more a week, have poor health and sleep, and drink alcohol (*p* = 0.001, *p* = 0.000, *p* = 0.014, *p* = 0.040, and *p* = 0.000, respectively). In contrast, regular smokers were less likely to have GHQ 12 scores ≥4 and to attend smoking cessation education (*p* = 0.017 and *p* = 0.035, respectively). The mean GHQ 12 score was significantly lower among regular smokers that among non-current smokers (*p* = 0.003) and in men than in women (*p* = 0.023).

**Table 3:** Assessment of smoking habits according to behavioral variables and mental health

	Non-current smokers (%)	Regular smokers (%)	*χ* ^2^	*p*-value
Residence				
With family	53 (13.2)	2 (3.8)		
Alone	348 (86.8)	50 (96.2)		
			3.789	0.052^a^
Skipping breakfast				
Less than twice a week	233 (58.1)	17 (32.7)		
Twice or more a week	168 (41.9)	35 (67.3)		
			12.02	0.001^a^
Eating between meals				
Less than once a week	252 (62.8)	33 (63.5)		
Once or more a week	149 (37.2)	19 (36.5)		
			0.008	0.931^a^
Eating out				
Less than twice a day	265 (66.1)	19 (36.5)		
Twice or more a day	136 (33.9)	33 (63.5)		
			17.181	0.000^a^
Health status				
Good	351 (87.5)	39 (75.0)		
Poor	50 (12.5)	13 (25.0)		
			6.037	0.014^a^
Physical activity,				
30 min or more a day	216 (53.9)	30 (57.7)		
Less than 30 min a day	185 (46.1)	22 (42.3)		
			0.272	0.602^a^
Participation in school club activities				
Yes	333 (83.0)	44 (84.6)		
No	68 (17.0)	8 (15.4)		
			0.082	0.775^a^
Sleeping status				
Good	307 (76.6)	33 (63.5)		
Poor	94 (23.4)	19 (36.5)		
			4.217	0.040^a^
Sleeping duration				
< 6 h	241 (60.1)	25 (48.1)		
≥ 6 h	160 (39.9)	27 (51.9)		
			2.745	0.098^a^
Alcohol drinking				
Never	209 (52.1)	8 (15.4)		
Less than three times a week	143 (35.7)	21 (40.4)		
Three times or more a week	49 (14.2)	23 (44.2)		
			42.928	0.000^a^
GHQ 12 score				
mean ± SD				
Female (*n* = 200)	3.67 ± 2.94	3.00 ± 4.63	–	0.621^c^
Male (*n* = 253)	2.94 ± 2.72	2.11 ± 2.07	–	0.023^c^
Total (n = 453)	3.30 ± 2.86	2.19 ± 2.35	–	0.003^c^
< 4	240 (59.9)	40 (76.9)		
≥ 4	161 (40.1)	12 (23.1)		
			5.684	0.017^a^
Received training in smoking cessation				
Yes	389 (97.0)	47 (90.4)		
No	12 (3.0)	5 (9.6)		
			5.59	0.035^b^

*GHQ* General Health Questionnaire (high scores indicate low mental health status), *SD* standard deviation. ^a^Chi-square test, ^b^Fisher’s exact test, ^c^*t* test

Table 4 summarizes the assessment of smoking habits according to the family history of smoking. Smoking was associated significantly with fathers’ smoking habits (*p* = 0.010).

**Table 4:** Assessment of smoking habits according to family members’ smoking

	Non-current smokers (%)	Regular smokers (%)	*χ* ^2^	*p*-value
Father				
Non-smoker or no father	330 (82.3)	35 (67.3)		
Regular smoker	71 (17.7)	17 (32.7)		
			6.605	0.010^a^
Mother				
Non-smoker or no mother	383 (95.5)	47 (90.4)		
Regular smoker	18 (4.5)	5 (9.6)		
			2.51	0.167^b^
Brother				
Non-smoker or no brother	371 (92.5)	45 (86.5)		
Regular smoker	30 (7.5)	7 (13.5)		
			2.195	0.172^b^
Sister				
Non-smoker or no sister	396 (98.8)	51 (98.1)		
Regular smoker	5 (1.2)	1 (1.9)		
			0.161	0.521^b^

^a^Chi-square test, ^b^Fisher’s exact test

Table 5 summarizes the professional responsibility results. Responses to five questions differed significantly between regular smokers and non-current smokers. The mean professionalism score was significantly lower among regular smokers than among non-current smokers (*p* < 0.001). Overall, 50.1% of the non-current smokers and 71.2% of the regular smokers responded that patients’ chances of quitting smoking were not increased if a dentist advised them to quit.

**Table 5:** Responses of dental students to smoking intervention statements

	Non-current smokers (%)	Regular smokers (%)	*χ* ^**2**^	*p*-value
1. Do you think that dental students should not smoke?				
Yes (score 1)	333 (83.0)	31 (59.6)		
No (score 0)	38 (9.5)	18 (34.6)		
No opinion (score 0)	30 (7.5)	3 (5.8)		
			26.861	0.000^a^
2. What is your view on smoking by patients?				
They should not smoke. (score 1)	245 (61.1)	22 (42.3)		
There is no problem with them smoking. (score 0)	38 (9.5)	8 (15.4)		
Patients may smoke at their discretion. (score 0)	109 (27.2)	20 (38.5)		
No opinion (score 0)	9 (2.2)	2 (3.8)		
			6.883	0.076^a^
3. We should serve as role models for their patients and the public.				
Yes (score 1)	369 (92.0)	44 (84.6)		
No (score 0)	32 (8.0)	8 (15.4)		
			3.135	0.113^b^
4. Dentists should master the ability to be non-smokers.				
Yes (score 1)	340 (84.8)	31 (59.6)		
No (score 0)	61 (15.2)	21 (40.4)		
			19.675	0.000^a^
5. Dentists should be examples to patients with regard to cessation of smoking.				
Yes (score 1)	329 (82.0)	32 (61.5)		
No (score 0)	72 (18.0)	20 (38.5)		
			19.96	0.001^a^
6. Dentists should advise patients not to smoke.				
Yes (score 1)	329 (82.0)	40 (76.9)		
No (score 0)	72 (18.0)	12 (23.1)		
			0.799	0.371^a^
7. Dentists should provide patients with information related to non-smoking.				
Yes (score 1)	376 (93.8)	45 (86.5)		
No (score 0)	25 (6.2)	7 (13.5)		
			3.662	0.077^b^
8. Are a patient’s chances of quitting smoking increased if a dentist advices him/her to quit?				
Yes (score 1)	200 (49.9)	15 (28.8)		
No (score 0)	201 (50.1)	37 (71.2)		
			8.163	0.004^a^
9. Dentists are free to smoke as people in other occupations.				
Yes (score 0)	128 (31.9)	26 (50.0)		
No (score 1)	273 (68.1)	26 (50.0)		
			6.706	0.010^a^
Professionalism score (0–9 points)				
mean ± SD	6.96 ± 1.99	5.64 ± 2.59	–	0.001^c^

*SD* standard deviation. ^a^Chi-square test, ^b^Fisher’s exact test. ^c^*t* test

Table 6 shows the results of the logistic regression analysis. The multivariate analysis revealed that male gender (OR = 5.449, *p* = 0.002, 95% CI = 1.851–16.040); higher academic year, including years 4 (OR = 14.923, *p* = 0.043, 95% CI = 1.094–203.531), 5 (OR = 21.935, *p* = 0.017, 95% CI = 1.746–275.497), and 6 (OR = 21.971, *p* = 0.018, 95% CI = 1.686–286.290); eating out twice or more a day (OR = 2.492, *p* = 0.019, 95% CI = 1.165–5.331); and drinking alcohol less than three times a week (OR = 3.336, *p* = 0.017, 95% CI = 1.241–8.970) or three times or more a week (OR = 9.484, *p* = 0.000, 95% CI = 3.335–26.970) were associated with higher odds of regular smoking. In contrast, GHQ 12 score ≥ 4 was associated with lower odds of regular smoking (OR = 0.339, *p* = 0.020, 95% CI = 0.136–0.845). For every point increase in the professionalism score, the odds of smoking decreased by a factor of 0.719 (*p* = 0.000, 95% CI = 0.608–0.850).

**Table 6:** Multiple logistic regression analysis for predictors associated with regular smoking

Independent variables	Category	Adjusted Odds ratio (95% CI)	*p*-value	Score Assigned
Gender	Female	1	–	0
	Male	5.449 (1.851–16.040)	0.002	1
Age (years)	18–20	1	–	0
	21–23	0.735 (0.161–3.362)	0.691	1
	24–26	0.481 (0.088–2.626)	0.398	2
	27≤	2.123 (0.396–11.382)	0.380	3
Academic year	1st	1	–	0
	2nd	5.002 (0.487–51.376)	0.176	1
	3rd	8.478 (0.716–100.318)	0.090	2
	4th	14.923 (1.094–203.531)	0.043	3
	5th	21.935 (1.746–275.497)	0.017	4
	6th	21.971 (1.686–286.290)	0.018	5
Skipping breakfast	Less than twice a week	1	–	0
	Twice or more a week	1.806 (0.848–3.846)	0.126	1
Eating out	Less than twice a day	1	–	0
	Twice or more a day	2.492 (1.165–5.331)	0.019	1
Health status	Good	1	–	0
	Poor	2.248 (0.855–5.911)	0.101	1
Sleeping status	Good	1	–	0
	Poor	1.232 (0.527–2.883)	0.630	1
Alcohol drinking	Never	1	–	0
	Less than three times a week	3.336 (1.241–8.970)	0.017	1
	Three times or more a week	9.484 (3.335–26.970)	0.000	2
GHQ 12 score	< 4	1	–	0
	≥4	0.339 (0.136–0.845)	0.020	1
Received training in smoking cessation	Yes	1	–	0
	No	2.985 (0.640–13.915)	0.164	1
Father’s smoking	Non-smoker or no father	1	–	0
	Regular smoker	1.812 (0.757–4.340)	0.182	1
Professionalism score (per 1 point increase)		0.719 (0.608–0.850)	0.000	–

-2 Log likelihood = 201.673

Hosmer and Lemeshow test: *χ*^2^ = 3.596, *p* = 0.892

Cox-Snell *R*^2^ = 0.235

Nagelkerke *R*^2^ = 0.461

*CI* confidence interval, *GHQ* General Health Questionnaire (high score indicate low mental health ststus)

## Discussion

This study showed that the overall prevalence of regular smoking among the respondents in our study was lower than reported for dental students in other countries [13, 29–35]. Consistent with previous studies, regular smoking was more prevalent among men than among women [13, 29–35]. Previous studies have indicated that the harmful effects of smoking during pregnancy on maternal and fetal health are well recognized among women in Japan and the United States [36, 37]. It is likely that female students have a better understanding and care more about pregnancy and childbirth as well as their own health than male students. Several studies have indicated that family influence is a major risk factor for and predictor of the initiation of smoking [13, 16], although one study showed that a family history of smoking was not associated with smoking. We found that 50 (96.2%) regular smokers did not live with their families. A previous study indicated that cigarette smoking in college students is often a social activity associated with facilitation of social interactions, to avoid feeling alone, and to behave as a member of a group [38]. Students may have become regular smokers because of the lack of family members who cautioned against smoking, and instead were influenced by their friends who were smokers. Many studies have shown that alcohol consumption is significantly associated with cigarette smoking [16, 39, 40], which was consistent with our findings. Alcohol is an effective anxiolytic in humans and animals [41, 42]; therefore, the desire to drink may be motivated by the wish to alleviate stress [43]. Another study indicated that drinkers had many other drinkers in their social network and the frequency of individual drinking was related to the frequency of drinking by others [44]. This trend was very similar to that seen in smokers [45]. These findings are correlated with the observation that eating out more times per day is associated with higher odds of being a regular smoker. Drinking and smoking may play roles in stress relief as well as in building and maintaining interpersonal relationships.

Recent studies have revealed very strong associations between cigarette smoking and mental health variables in college students in the United States [46, 47]. In contrast, we found that the mean GHQ 12 score was significantly lower among regular smokers than among non-current smokers, suggesting that the mental health state was lower among non-current smokers than among regular smokers. In a recent study, the mean GHQ 12 scores for female and male participants were 3.82 and 3.00, respectively, and significant gender-related differences were observed among Japanese medical students regardless of smoking habit [48]. Consistent with these findings, mean GHQ 12 scores were higher in women than in men in this study. As 90.4% of regular smokers were men, it is natural that the mean GHQ scores of all regular smokers were close to those of regular male smokers. However, the mean GHQ scores among regular smokers were lower than those among non-current smokers, especially in men. Therefore, smoking appeared to have some impact on the decline in GHQ score. Another study suggested that nicotine critically regulates brain areas that are involved in the inhibition of negative emotions, such as anger [49], which may be one of reason for the lower scores among regular smokers than non-current smokers.

Univariate analyses showed that the prevalences of skipping breakfast, poor health status, and poor sleep habits were significantly associated with regular smoking, although they were not statistically significant on multivariate analysis. These findings suggested that regular smokers tended to have unhealthy lifestyles, including nocturnal habits. Generally, there are large variations in the preferred timing of sleep and activity, and this temporal directionality of activities of individuals is referred to as the chronotype [50]. In 1976, Horne and Ostberg [50] developed a questionnaire to classify individuals based on their preferences for sleep timing and daily performance, and classified chronotype into three types: evening type, morning type, and intermediate type. Evening type is associated with peak performance toward the end of the day. Unfortunately, in modern society sleep timing, especially on work days, is influenced by social norms, a phenomenon known as “social jet lag” [51]. Obviously, this is disadvantageous in evening types. Many studies have indicated lifestyle-related health problems in evening type people. Evening types were reported to skip breakfast more often than morning types [52]. Evening types experienced anxiety and negative moods, tended to have lower self-esteem, performed worse in school, and were suggested to be more susceptible to stress [53]. Furthermore, evening types with reduced sleep on weekdays compared to weekends showed higher incidences of depressive moods [54] and engaged in more negative health behaviors than morning types [55]. In this study, we did not diagnose the chronotypes of the students, although the behavioral features associated with regular smokers on univariate analyses were consistent with evening types. Only mental health status in regular smokers was inconsistent with the characteristics of evening types. However, our results are reasonable if students with poor mental health status due to social jet lag used smoking to improve their condition. Students who are aware that they have a predisposition for the evening type chronotype, especially men, must manage their health to a greater degree than others because they have the potential to become regular smokers.

We asked the students nine questions associated with professional responsibility, including their intention to counsel smokers about quitting, and found significantly lower scores among regular smokers compared with non-current smokers. These findings suggest that regular smokers are less conscious health professionals. In addition, 50.1% of the non-current smokers and 71.2% of the regular smokers responded that patients’ chances of quitting smoking are not increased if a dentist advises them to quit. The Global Health Professions Student Survey of 28,420 medical, dental, nursing, and pharmacy students from eight countries showed that dental students had significantly lower odds of being taught to provide educational materials to support smoking cessation for patients who want to quit smoking, compared with medical, nursing, and pharmacy students [56]. We also found that more than half of the students had an inadequate attitude toward smoking cessation by their patients. Prakash et al. [57] demonstrated that dentists can play an important role in primary prevention of adverse health effects by promoting the cessation of tobacco use among their patients.

In the clinical training of dental students, information about tobacco cessation treatment is provided by various educational methods, such as didactic lectures, web-based learning, and interactive CD-ROM programs. In addition, Objective Structured Clinical Examinations as an assessment method in clinical training was provided [58, 59]. Singleton et al. [60] suggested that a method that combined lectures with practice sessions with standardized patients increased participants’ subjective norms, perceived skills, and intentions to provide tobacco cessation treatment more than lectures alone. Therefore, it is necessary to apply more practical learning and assessment after passive learning during training of students to support smoking cessation for their patients.

We found that Japanese dental students have inadequate attitudes and skills to advise their patients to quit smoking, although most students held positive attitudes toward smoking prevention and cessation. The development of educational programs including tobacco cessation treatment for dental students in Japan is necessary.

## Conclusions

Regular smoking was strongly associated with male gender, higher academic year, consumption of alcohol, and eating out more often. Mental health status among regular smokers was better than among non-current smokers. Behavioral features of regular smokers were consistent with the characteristics of evening types. Students who are aware that they have predisposition for the evening type chronotype, especially among men, must manage their health much more carefully than those with other chronotypes. Furthermore, we found that more than half of the dental students had inadequate attitudes and skills to advise their patients to quit smoking. Consequently, the development of educational programs for dental students in Japan is necessary.

## Abbreviations

AGFI: adjusted goodness-of-fit index
CFI: comparative fit index
CI: confidence interval
GFI: goodness-of-fit index
GHQ: General Health Questionnaire
OR: odds ratio
RMSEA: root-mean-square error of approximation
SD: standard deviation
